# Fluorescence anisotropy of diphenylhexatriene and its cationic Trimethylamino derivative in liquid dipalmitoylphosphatidylcholine liposomes: opposing responses to isoflurane

**DOI:** 10.1186/2046-1682-5-5

**Published:** 2012-03-24

**Authors:** Steven C Nelson, Steven K Neeley, Eric D Melonakos, John D Bell, David D Busath

**Affiliations:** 1 Department of Physiology and Developmental Biology, Brigham Young University, Provo, UT 84602 USA; 2 WIDB 574, Dept. of Physiology and Developmental Biology, Brigham Young University, Provo, UT 84602, USA; 3 The University of Toledo College of Medicine, 3045 Arlington Avenue, Mail Stop 1043, Toledo, OH 43614, USA; 4 University of Texas Southwestern, 5323 Harry Hines Blvd., Dallas, TX 75390-9003, USA; 5 Dept. of Bioengineering, Univ. of Utah, Salt Lake City, UT 84112, USA; 6 Dean of Undergraduate Education, Brigham Young University, Provo, UT 84602, USA

## Abstract

**Background:**

The mechanism of action of volatile general anesthetics has not yet been resolved. In order to identify the effects of isoflurane on the membrane, we measured the steady-state anisotropy of two fluorescent probes that reside at different depths. Incorporation of anesthetic was confirmed by shifting of the main phase transition temperature.

**Results:**

In liquid crystalline dipalmitoylphosphatidylcholine liposomes, isoflurane (7-25 mM in the bath) increases trimethylammonium-diphenylhexatriene fluorescence anisotropy by ~0.02 units and decreases diphenylhexatriene anisotropy by the same amount.

**Conclusions:**

The anisotropy data suggest that isoflurane decreases non-axial dye mobility in the headgroup region, while increasing it in the tail region. We propose that these results reflect changes in the lateral pressure profile of the membrane.

## Background

Inhaled volatile general anesthetics block pain, memory, consciousness, and movement by inhibiting excitatory channels and activating inhibitory channels [1] with weak potency (generally > 100 μM inhibitory constant). Debate continues over whether the mechanism is lipid mediated [2,3] or involves direct binding to ion channels [4], and obviously could involve both. In molecular dynamics simulations, lateral pressures have been found to be positive in the head and tail regions and very negative at the level of the glycerol ester oxygens [5]. Insertion of surfactants, such as volatile anesthetics, could influence these pressures [2] by increasing area per headgroup, thinning the membrane to maintain molecular volume, and increasing the lateral pressure in the headgroup and glycerol regions while reducing it in the tail region. Indeed, ethanol was found in simulations to increase the lateral pressure in the glycerol region [6,7]. Halothane was found in simulations to accumulate below the interface [8]. Such changes may differentially affect protein cross-section during function [9], and have been suggested to play a role in modulation of ion channels [10-12].

Support for the theory that general anesthetics affect the lateral pressure profile has been provided by the observation that diphenylhexatriene (DPH) and trimethylammonium-DPH (TMA-DPH) show decreased fluorescence anisotropy with increased volatile anesthetic addition in red blood cell ghosts [13]. This begs interpretation from molecular dynamics simulations, both regarding the impact of anesthetics on lateral pressure profile, and of lateral pressure profile on the position and dynamics of the dyes related to anisotropy changes. However, complex lipids are difficult to simulate adequately, so it is important to obtain similar measurements in purified lipid systems. We chose to focus on dipalmitolyphosphatidylcholine (DPPC) because of its readily accessible phase transition. Introduction of lipids with high microscopic curvature (dioleoylphosphatidylethanolamine) into dioleoylphosphatidylcholine bilayers was interpreted to influence lateral pressure profile in one study based on pyrene-labeled-lipid excimer formation as a probe [14]. However, we chose to use the DPH probes used by Norman et al. [13] with the intention that they should respond to lateral pressure changes at different levels without the added complexity that the probes are bound together at different tether lengths. Surprisingly, in DPPC, we observed a crossover phenomenon for TMA-DPH: an increase in anisotropy induced by isoflurane. Being charged, this dye is known to float higher in the bilayer leaflet [15]. This phenomenon might be expected in other alkyl fatty acid lipids (like sphyngomyelin) and should be a useful target for future molecular dynamics simulations. Although the results do not speak to whether ion channels would be affected at relevant concentrations, they do correlate well with Cantor's basic premise [9].

## Results

The effects of isoflurane in the tail and head regions of DPPC LUVs were assessed using DPH and the charged analog TMA-DPH respectively.

**Figure 1:**
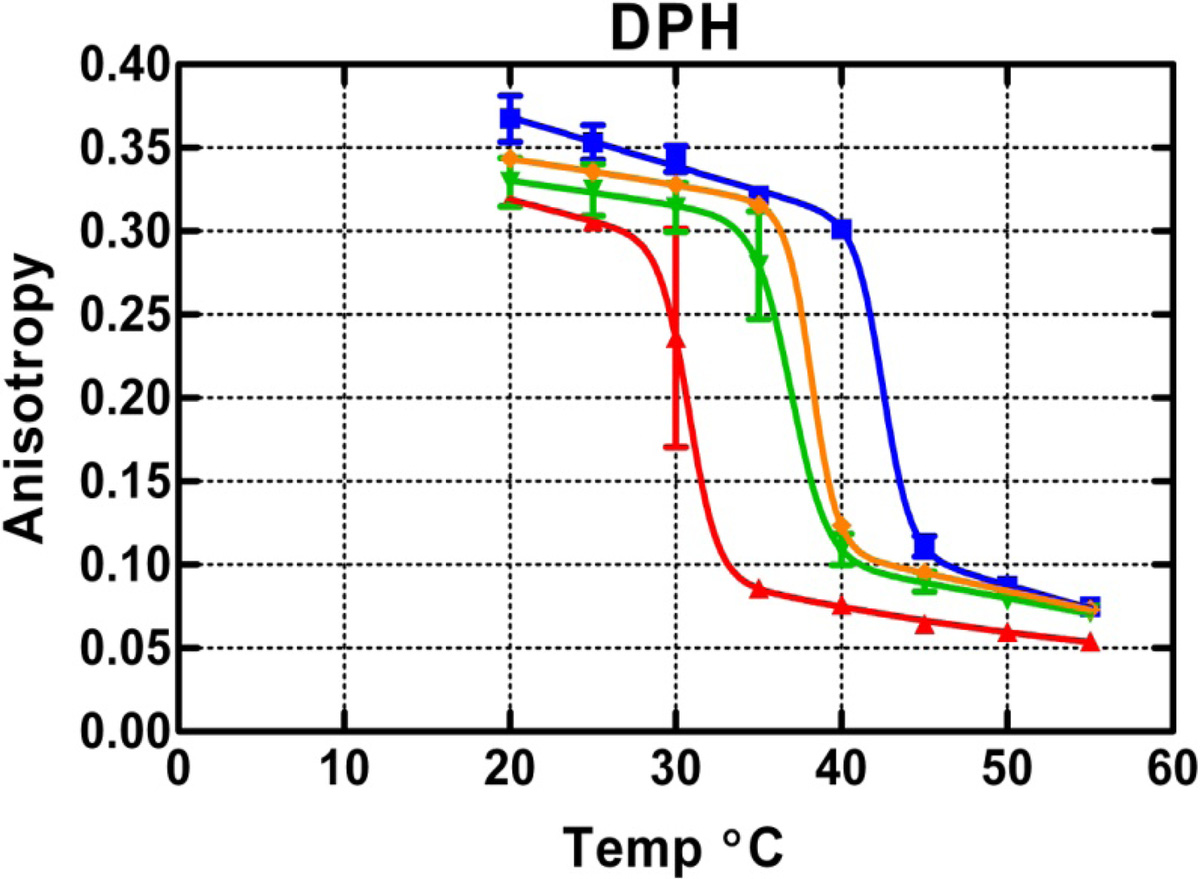
**Mean DPH anisotropy in DPPC LUVs measured in 5°C increments from 20-55°C at isoflurane concentrations of 26.3 (*red triangles*), 15.3 (*inverted green triangles*), 7.6 (*orange diamonds*) and 0 mM (*blue squares*)**. Data points indicate mean ± 1 SE. Theoretical curves are based on equation 1.

Figure 1 shows the calculated mean anisotropy for DPH. Control samples revealed a mean primary phase transition between 40 and 45°C in the absence of anesthetic, with a fitted parameter of 42.5°C. A downward shift in this phase transition of 4.2, 5.5 or 12°C was observed when samples were prepared containing isoflurane at concentrations of 7.6, 15.3, or 26.3 mM. In the gel state, there was a significant downward trend in anisotropy with increasing isoflurane concentration (*p *< 0.03 by linear regression), consistent with increasing the non-axial motion of the fluorophore. Likewise, isoflurane reduced the anisotropy in the liquid phase, with the effect being strongest at 26.3 mM (effects of temperature, *p *< 0.0001, and isoflurane, *p *= 0.0007, were both significant without interaction, *p *= 0.13, by two-way ANOVA).

**Figure 2:**
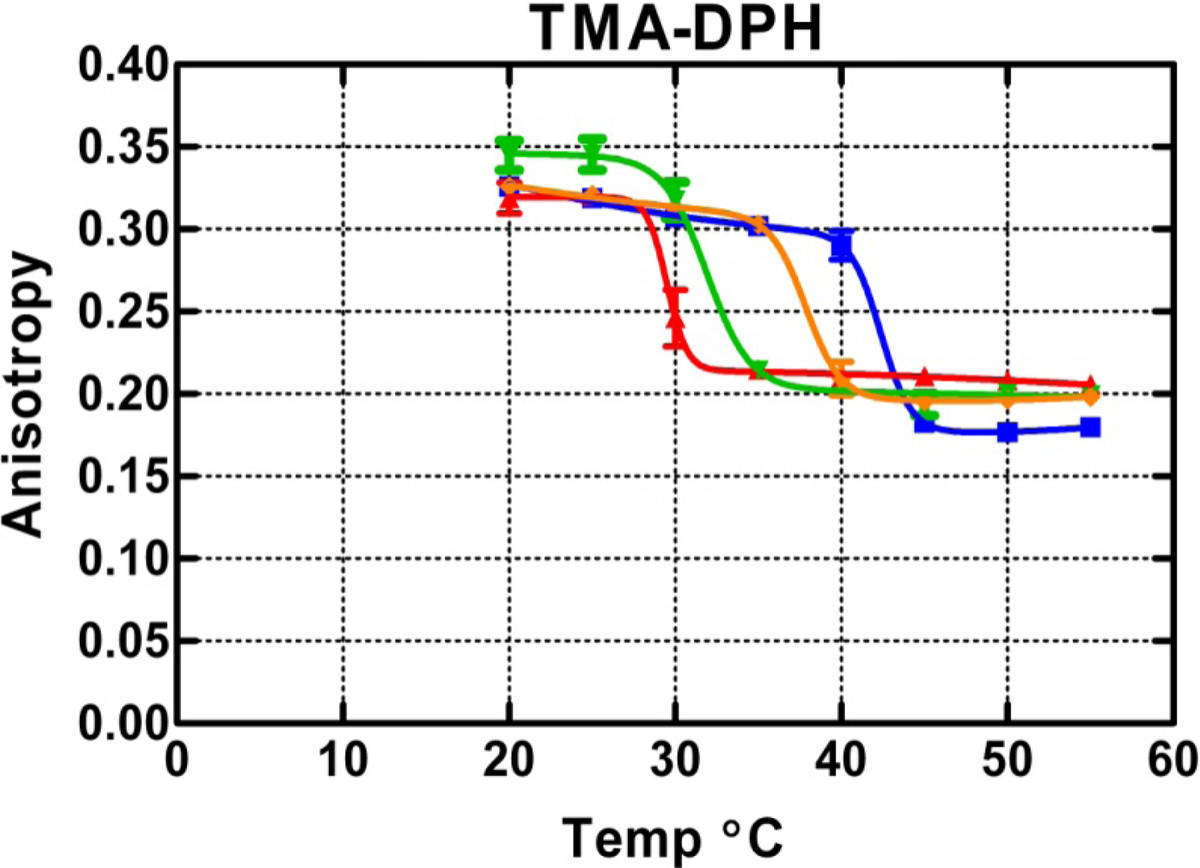
**Mean TMA-DPH anisotropy under the same conditions as Figure 1**.

Figure 2 shows that TMA-DPH sensed similar downward shifts in the phase transition temperature when isoflurane was added. In contrast, in the liquid-crystalline state, a striking difference was observed. Instead of causing a reduction of the anisotropy, the anesthetic caused an increase in anisotropy such that the test curves cross the control curve at ~43°C. The non-monotonic behavior for the gel state was somewhat variable (insignificant by regression but differences with concentration marginally detectable by two-way ANOVA, *p *= 0.04; the effect of temperature and the interaction between temperature and isoflurane concentration were not significant, *p *> 0.1), but the crossover with matched controls occurred in every experiment. The anisotropy change in the liquid-crystalline state appeared to be most sensitive to anesthetic, because it was maximal at the lowest concentration, 7.6 mM (*p *= 0.0005 by two-way ANOVA; effects of temperature and interaction were not significant, *p *> 0.2). These phenomena were confirmed with an additional independent experiment at 15.3 mM isoflurane (data not shown).

**Figure 3:**
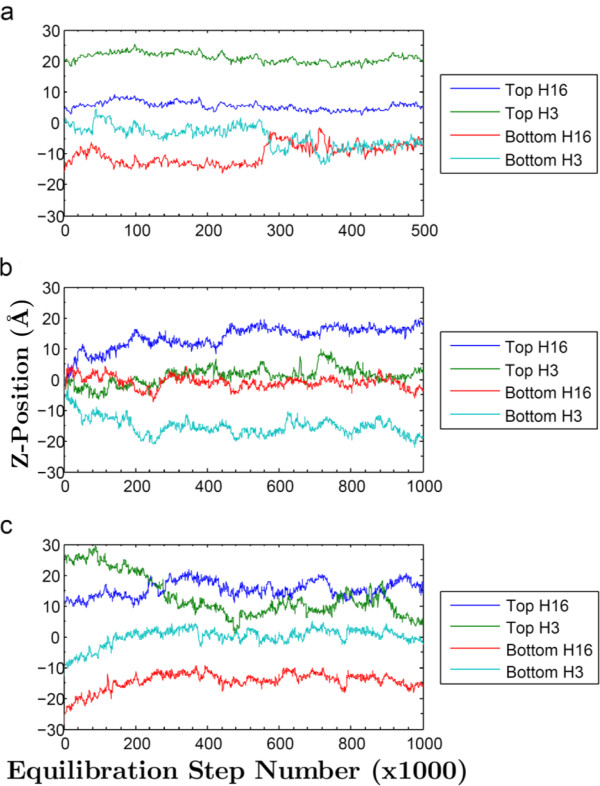
**Z-Axis Positions of DPH Hydrogen Atoms**. Z-positions of the DPH end hydrogen atoms throughout three different molecular dynamics runs, each with different initial conditions. a) "Inserted"; b) "Sandwiched"; c) "Halfway".

**Figure 4:**
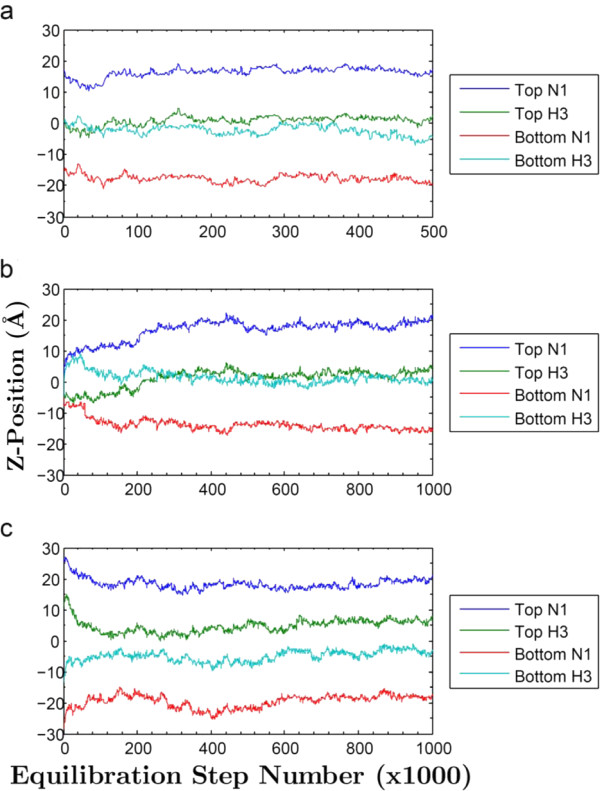
**Z-Axis Positions of TMA-DPH Nitrogen and Hydrogen Atoms**. Z-positions of the TMA-DPH nitrogen atom and the opposing end's hydrogen atom. a) "Inserted"; b) "Sandwiched"; c) "Halfway". An extra 1.9 Å in the z-direction should be factored in when considering the additional length of the nitrogen to its methyl hydrogens. Simulation parameters were identical to the DPH runs, with the exception of six chloride atoms being used instead of four.

Molecular dynamics trajectories were generated using three dramatically different starting configurations for bilayers containing either two DPH or two TMA-DPH molecules. In the first starting configuration, the two dye molecules were inserted into opposing leaflets each replacing a lipid molecule. In the second, they were positioned parallel to the bilayer, rather than perpendicular, in the center between the two leaflets. In the third, each dye molecule was pushed into the leaflet but still projecting out into the water, perpendicular to the bilayer. In the first case, the dye molecules remained approximately stable throughout the simulation (Figures 3a and 4a), vertical in their starting leaflets (with the exception of the DPH in the lower leaflet in Figure 3a, which rotates into a horizontal position at 300,000 steps, i.e. 600 ps, and remains at Z = 7-8 Å for the remainder of the run). Remarkably, in the second and third cases they attained similar positions during the heating and initial 500,000 steps (1.0 ns) of simulation (Figures 3b, c and 4b, c). In all three cases, the dye molecules rapidly attained and usually persisted in a position perpendicular to the bilayer, with one dye molecule per leaflet. The DPH molecules left the sandwiched positions (Figure 3b) as rapidly as the TMA-DPH (Figure 4b), i.e. within ~100,000 steps (200 ps).

The average z-positions from the last halves of the simulations were used to determine the positions of the dye molecules in the leaflets. Table 1 shows the average z-positions of the terminal hydrogens for each of the DPH two molecules in each of the three starting configurations and Table 2 shows the average z-positions of the TMA nitrogen and the terminal hydrogen for the TMA-DPH molecules. The standard deviations between frames are modest for each case, whereas the differences between starting configuration sets are somewhat larger, demonstrating that dye configurational space is not thoroughly sampled on the ns time scale, even though the dye molecules attained their final positions on the sub-ns time scale. For each dye, the results represent six unique approaches, from six unique starting points, to possible metastable configurations. All of the dye molecules initially achieved approximately the same configuration, i.e. contained within one leaflet, perpendicular to the bilayer, although there were indications of flexibility, at least for DPH, in both the movement of the lower leaflet dye to the horizontal position in Figure 3a and also the temporary visits to horizontal states seen at steps 240 K, 600 K, and 850-900 K in the upper leaflet dye in Figure 3c. The trajectory frames therefore represent a modest sampling of all such metastable configurations. The average atom z-positions for each configuration were averaged as a small-sample representation of the ensemble of configurations. From the six different DPH configurations, the average z-position of the terminal hydrogen atoms near the interfaces averaged 14.9 ± 4.0 Å and those nearest the center of the bilayer averaged 4.2 ± 3.5 Å. For TMA-DPH, the TMA nitrogen always resides near the interface, at average z-positions of 17.4 ± 1.4 Å, with the terminal hydrogens on the DPH group near the bilayer center at average z-positions of 2.8 ± 1.9 Å. The larger standard deviations for DPH demonstrate the flexibility in positioning shown by two of the DPH molecules.

**Table 1:** Means and standard deviations for the z-axis positions of the end hydrogen atoms of the dyes nearest to the lipid-water interface and the center of the bilayer are listed

	Top DPH		Bottom DPH	
**Run Type**	**Interface**	**Center**	**Interface**	**Center**

*Inserted (N = 250)*	20.03 ± 1.06	4.67 ± 0.90	-7.92 ± 2.60	-67 ± 2.98

*Sandwiched (N = 500)*	16.17 ± 1.25	2.24 ± 2.29	-16.06 ± 2.05	-13 ± 1.45

*Halfway (N = 500)*	15.61 ± 2.14	9.54 ± 2.92	-13.82 ± 1.59	0.65 ± 1.61

Mean ± S.D., (N = 6) of |z_near-interface H_|			14.9 ± 4.0	

Mean ± S.D., (N = 6) of |z_near-center H_|			4.2 ± 3.5	

Statistics are from the last half of each DPH run.

**Table 2:** Mean and standard deviation for z-axis positions of nitrogen and hydrogen atoms from the last half of each TMA-DPH run

Top TMA-DPH			Bottom TMA-DPH	
**Run Type**	**N1**	**H3**	**N1**	**H3**

*Inserted (N = 250)*	17.12 ± 0.86	1.45 ± 0.77	-17.82 ± 1.10	-2.65 ± 1.81

*Sandwiched (N = 500)*	18.09 ± 1.13	2.39 ± 1.02	-14.78 ± 0.93	0.29 ± 1.07

*Halfway (N = 500)*	18.01 ± 1.20	5.75 ± 1.25	-18.78 ± 1.34	-4.13 ± 1.53

Mean ± S.D., (N = 6) of |z_N1_|			17.4 ± 1.4	

Mean ± S.D., (N = 6) of |z_H3_|			2.8 ± 1.9	

## Discussion

Isoflurane decreases the membrane transition temperature, similar to previous observations [16,17]. This indicates that isoflurane is more soluble in the liquid than the gel phase, and would be consistent with the concept that tail separation by headgroup intercalation of the anesthetic molecules facilitates liquefaction. Both dyes track this change, and, although the temperature resolution in these experiments is too low to allow analysis, it is clear that it is strongly dependent on isoflurane concentration.

Steady-state anisotropy represents the average limitation to non-axial movements (wobble of the rod-shaped molecule in a conical or hour-glass shaped cage) during the random interval between photon absorption and fluorescence emission [15]. It can be used to estimate the free volume available to the probe, which in turn can be related to lateral pressure at the level of the probe [18]. Currently, this relationship between fluorescence anisotropy and lateral pressure profile needs further refinement, especially through molecular dynamics simulations. The model system employed here and results should spur further definition of this relationship.

Fluorescence quenching experiments [19] with dioleoylphosphatidylcholine bilayers have been used to estimate the position of DPH within the membrane. These experiments are likely to be relevant to DPPC in the liquid state based on similar bilayer thicknesses - phosphorus separation of 40.3 Å for dioleoylphosphatidylcholine [20], and 39.2 Å at 50°C for DPPC [21]. The quenching experiments revealed that the DPH molecule is positioned approximately parallel to the lipid molecules in the tail region of one leaflet of the bilayer, similar to simulation results obtained previously [22], with its center 7.8 Å from the bilayer midplane. The terminal carbons are positioned 1.3 and 14.3 Å from the bilayer center. The central carbon of the DPH moiety in TMA-DPH molecules is located 3.1 Å closer to the bilayer surface, with the terminal carbon 4.4 Å from the center of the bilayer and the TMA N 18.7 Å from the center of the bilayer. These results are consistent with the molecular dynamics trajectories reported here: the dye has a strong propensity to orient in one leaflet with one end near the bilayer center and the other near the interface. Averaging among the three initialization cases, the N of TMA-DPH is located 17.4 Å from the bilayer center. Likewise, the terminal hydrogens near the bilayer center are in the same leaflet and within a few Å of the center for both dye molecules. Given the lengths of the molecules, the average tilt angles (17.5° [15]), and the bilayer thickness, we estimate that TMA-DPH anisotropy reflects the lateral pressures in all three regions (headgroup, glycerol backbone, and tail), whereas DPH should be sensitive to the lateral pressure primarily in the tail region.

Our simulations address controversies that exist over the position of DPH in lipid bilayers [23]. Neutron diffraction studies [24] contradict the conclusion of the fluorescence quenching parallax studies [19], indicating the DPH partitions into the center of the bilayer, between the two leaflets. However, for the neutron diffraction, it was necessary to use 50× higher dye:lipid ratio than is usually used in fluorescence experiments like those reported. Given the alacrity with which sandwiched dye molecules abandon the center of the bilayer in our simulations, it seems likely that virtually no dye is sandwiched between leaflets at low mole ratios. The high dye concentrations used, (and perhaps configurational effects of high headgroup spacing due to the 75% humidity conditions used [25]) may lead to the formation of a separated dye phase in the bilayer interior. Although some dye molecules can be positioned parallel to the lipid surface [26,27], these should only contribute modestly to the fluorescence anisotropy, being but a small component of the fluorescence amplitude, perhaps in part due to aqueous quenching [28].

**Figure 5:**
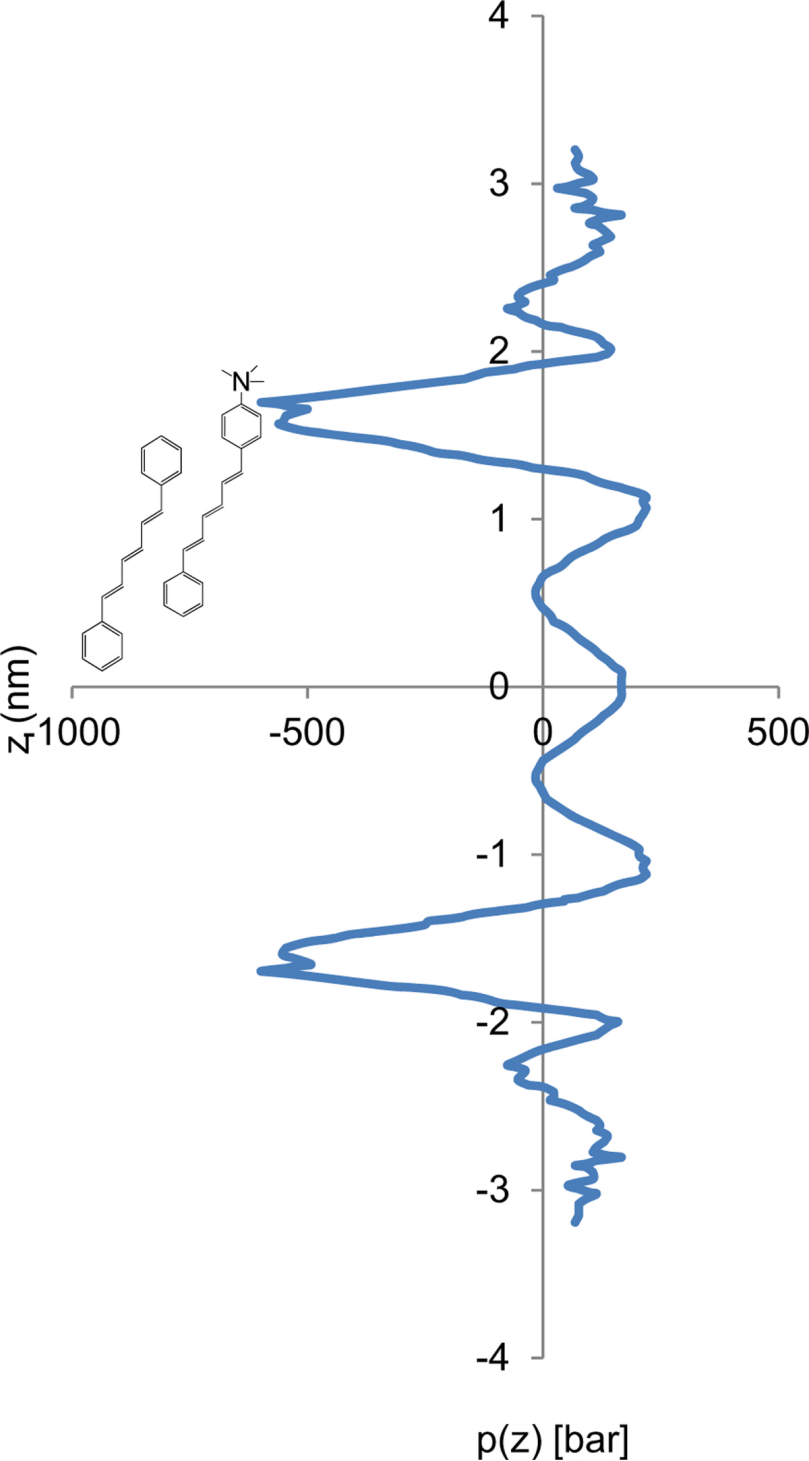
**Lateral pressure profile in DPPC as reported previously **[32]**, with the positions of DPH (left) and TMA-DPH (right), taken from fluorescence quenching parallax analysis and confirmed here and in **[22]** by simulation, accurately superimposed**.

Isoflurane increased the anisotropy of TMA-DPH in the liquid-crystalline phase, implying reduced fluctuations in the available cone angle, possibly due to increased lateral pressure. For DPH, the reduction of anisotropy caused by isoflurane indicates increased dye mobility in the lipid tail region. This decrease was observed at both low and high temperatures, indicating that, although the anesthetic must be more miscible with the liquid phase of the membrane (based on the leftward shift of the melting temperature), it must still partition sufficiently into the gel phase to perturb motion constraints on DPH. Presumably, formation of segregated complexes among isoflurane molecules or other heterogeneities account for the ability of the anesthetic to alter DPH anisotropy even though its miscibility in the DPPC gel phase is low. The phenomenon of lipophilic membrane contaminants altering both the phase transition and physical behavior of the phospholipid at temperature extremes has been observed previously for molecules such as fatty acids and lysophospholipids [29,30]. We suggest that the effect of isoflurane on the overall anisotropy may be due to separation of the headgroups by intercalated anesthetic molecules [31] and relief of lateral pressure in the tail region. Therefore, the increased anisotropy for TMA-DPH must be due to increased lateral pressure in the headgroup region, as would be expected for preferential absorption of anesthetic molecules to that region. The lateral pressure is negative (i.e. is lateral tension) at the level of the glycerol backbone due to the propensity of water molecules to flee the hydrophobic core. It is positive in the bilayer interior due to crowding imposed by the high-tension surface above and the loss of configurational entropy of the partially oriented tails. Extensive simulations done with a pure DPPC bilayer membrane in pure water at 323 K [32] yield the symmetrized lateral pressure profile redrawn in Figure 5 as a diagram to illustrate the size and positions of the dye molecules in the monolayers. The high tension trough between 1.2 and 1.9 nm from bilayer center represents the interface between the hydrophilic bath and the hydrophobic bilayer interior and may attract anesthetic molecules, which then modulate the profile and significantly affect dye oscillations.

These results can be contrasted and compared to two related studies. First, an extensive study of the effects of several volatile anesthetics on DPH and TMA-DPH anisotropy in red blood cell ghosts showed that all anesthetics caused anisotropy to decline in a dose-dependent fashion, not only for DPH, but also for TMA-DPH [13]. It must be acknowledged, therefore, that the crossover reported here may be unique to DPPC, or perhaps more generally to purified alkyl-tail lipids. Nevertheless, we note that sphingomyelin, a common component of rafts in neuronal cell membranes, has two alkyl tails like DPPC (as opposed to one alkene and one alkyl tail commonly found in natural non-raft membranes), so the crossover phenomenon may be pertinent to modulation of lateral pressure profiles in lipid rafts. As a side note, we point out that the results of Norman et al. [13] were interpreted as evidence against microviscosity changes playing a role in ion channel modulation by anesthetics. This was due to the lack of uniformity in anisotropy changes at MAC, the mean alveolar concentration required to immobilize half of human patients, for different anesthetics. However, in retrospect, it is noteworthy that in many cases there were abrupt shifts in the curve or the curvature of anisotropy vs. anesthetic concentration at or near MAC for one dye or the other, which may suggest exaggerated effects of individual anesthetics on lateral pressure profile near their MAC values. Such changes could affect the splay in helix bundle membrane proteins.

On the other hand, the results were reproducible and our interpretation from the TMA-DPH anisotropy changes in DPPC that isoflurane increases pressure (i.e. reduces the negative pressure, or the tension) in the glycerol backbone region while decreasing pressure in the tail region is consistent with results of excimer fluorescence. Using vesicles doped with di-pyrenyl phosphatidylcholine having tails of different lengths, 4, 6, 8, or 10 carbons [14], it was observed that pyrenyl groups held close to the membrane surface showed lower excimer formation upon addition of DOPE, while pyrenyl groups attached beyond the *cis *double bonds showed increased excimer formation upon addition of DOPE. This was interpreted to mean that DOPE changed the lateral pressure profile, decreasing pressure in the headgroup/backbone region and increasing pressure in the distal tail region. However, in that approach, different tail lengths changed both pyrenyl depth and confinement. The results presented here suggest that lateral pressure, and not just confinement, contributed to the DOPE effect in excimer formation.

More broadly, this project is intended to complement more general explorations into how anesthetics and other small amphiphilic compounds might influence ion channels and other membrane proteins indirectly by perturbing the pressure profile or other characteristics of lipid membranes. Previously studied examples include effects of microscopic curvature on alamethecin channel kinetics [33], effects of numerous classes of amphiphiles (e.g. lysolecithin [34], alcohols [35], and halothane [36]) on gramicidin channel properties, effects of short chain alcohols and general anesthetics on potassium channel conductance properties [37], effects of small organic acids on monolayer properties, tadpole immobilization, and glycine receptors [38], and effects of ketamine on measured bilayer thickness and simulated lateral pressure profile [39]. All of these studies indicate that amphiphiles can influence membrane mechanics, and possible membrane protein structure and function, when found in the bath at the 0.1-1 mM level, similar to MAC levels for volatile anesthetics.

It must be noted that the results reported here are limited in scope and utilize high isoflurane concentrations, and are therefore preliminary in nature. Only one lipid species was analyzed and both the durations of the molecular dynamics simulations and the equilibration periods for the anisotropy experiments are modest. In particular, the flexibility of the DPH molecules in the simulations requires more extensive simulations for complete analysis. For instance, simulations on the order of 20-50 ns would allow consideration of the full range of angles available to DPH during the excited state and therefore direct verification of the anisotropy under the various experimental conditions.

In the future, the effect of lipid species on the crossover effect should be examined more closely, as crossover was not observed in erythrocyte ghosts [13], and expansion of the time domain for both the anisotropy experiment and the simulations should be examined. A complete free energy profile for rotations of the dye in the leaflet and during passage through the inter-leaflet region would be interesting, particularly for DPH, which is not anchored by a charged group at one end like TMA-DPH. The impact of isoflurane and other volatile anesthetics on the lateral pressure profile and on the dynamics of the fluorophores could be explored by simulation. Preliminary work in our lab indicates that these will be fruitful directions.

## Conclusions

The current result shows differential effects of anesthetic on lateral pressure at two different depths in the bilayer using untethered probes. Specifically, isoflurane appears to elevate lateral pressure in the headgroup region of DPPC membranes and reduce it in the tail region.

## Materials and methods

### Materials

1,6-Diphenyl-1,3,5-hexatriene (DPH) and its cationic derivative 1-(4-trimethylammoniumphenyl)-6-phenyl-1,3,5-hexatriene *p*-toluenesulfonate (TMA-DPH) were obtained from Molecular Probes, now Invitrogen (Eugene, OR). Both were suspended in dimethylformamide (1 mM) and stored at 4°C. To prepare a saturate buffer, ~1 ml pure isoflurane (Hospira, Lake Forest, IL) was injected gently into a glass vial and then overlaid with ~5 ml buffer solution (150 mM NaCl, 20 mM HEPES at pH 7.0), capped, and allowed to equilibrate at 4°C for at least 24 hours to saturation. Assuming a solubility of 13.4 mM at 25°C [40,41], the concentration of isoflurane at 4°C in the saturated solution is estimated to be 30.5 mM using a temperature dependence of -4%/°C [42]. Dipalmitoylphosphatidylcholine, dissolved in chloroform at 10 mg/ml, was acquired from Avanti Polar Lipids (Birmingham, AL). All other reagents were obtained from standard sources.

### Sample preparation

Large unilamellar vesicles (LUVs) were formed using 2.72 μmol DPPC and 2.0 nmol DPH or TMA-DPH in 4.4 ml of a mixture of isoflurane-saturated and isoflurane-free buffer solutions to produce final isoflurane concentrations of 7.6, 15.3, or 26.3 mM. In each case, a parallel isoflurane-free control was prepared and measured. At this low lipid concentration lipid absorption should minimally perturb aqueous isoflurane concentration. For each case, a cuvette was completely filled with the solution, carefully capped to avoid isoflurane evaporation, and stirred for at least ten minutes prior to measurements.

### Fluorescence spectroscopy

Steady-state fluorescence anisotropy data were acquired using a PC-1 spectrophotometer (ISS; Champaign, IL), using Glan-Thompson polarizers and 16 nm bandpass monochrometers. For both DPH and TMA-DPH, excitation was set to 350 nm and emission was measured at 452 nm. Temperature was controlled by a circulating water bath, and sample homogeneity was maintained by magnetic stirring. Data were gathered and anisotropy calculated as described previously [43]. After equilibration for 10 minutes at each temperature, anisotropy was measured to high accuracy three times.

### Analysis

Non-linear least squares fits were obtained using a single phase-transition equation with a quadratic baseline:

(1) $$r\left(T\right)=\frac{r_{\text{max}}-r_{\text{min}}}{1+e^{n\left(T-T_{m}\right)}}+r_{\text{min}}+AT+BT^{2}$$

where T is temperature (°C), T_m _is the lipid melting temperature, *n *describes the cooperativity of the melting transition, r_min _is the high-temperature limit for anisotropy, r_max _is the low-temperature limit for anisotropy and A and B are arbitrary baseline parameters. The parameter n was poorly constrained with the sparse temperature sampling used, but was included for completeness. The slight downward drift of the baseline may reflect slow equilibration of the dye in the liposome membranes.

Differences in anisotropy values at the high and low temperatures surrounding the phase transitions were evaluated by two-way analysis of variance (ANOVA). The two parameters compared for main effects in the ANOVA were temperature and isoflurane concentration. Interaction between the two parameters was also estimated. Trends with isoflurane concentration were assessed by linear regression when the *p*-value for isoflurane effect in the ANOVA was less than 0.05.

### Molecular dynamics simulation

Using Chemistry at Harvard Macromolecular Mechanics (CHARMM) software Version 37, lipid bilayer dynamics trajectories with TMA-DPH and DPH in several initial positions in a DPPC bilayer were obtained. Systems were each composed of a bilayer of 128 lipid molecules (or 126 in the case of the "Inserted" runs) arranged in two 8 × 8 leaflets normal to the z-axis, 3633 water molecules, 4 sodium atoms, 4 chloride atoms (6 in the case of TMA-DPH, to compensate for the +1 overall charge of each dye), and 2 dye molecules. Simulations were performed using 2 fs steps, without constraints, with constant isotropic pressure of 1.0 Atm, with constant temperature of 330 K, with periodic boundary conditions in a tetragonal cell, and with particle mesh Ewald sums for long-range electrostatics interactions. Short range interactions were limited by default nonbonded cutoffs. After minimization the systems were heated for 3.3 ps and then simulated, taking the first half of the simulation as configurational and thermal equilibration periods. The first system consisted of two dye molecules, one in each leaflet, replacing one lipid molecule each ("Inserted", 1 ns total simulation time); the second consisted of two dye molecules, oriented perpendicular to the z-axis and sandwiched between the two leaflets ("Sandwiched", 2 ns total equilibration time); the last consisted of two dye molecules, each beginning with half of the molecule in the water (including the TMA portion for TMA-DPH runs) and half in the lipid region ("Half-way", 2 ns total equilibration time).

## Competing interests

The authors declare that they have no competing interests.

## Authors' contributions

SCN confirmed the experimental results, analyzed the data, and composed associated sections of the manuscript. SKN carried out the initial fluorescence anisotropy experiments. EM carried out and composed the section on molecular dynamics. JDB participated in the design of the study, performed the statistical analysis, and heavily revised the manuscript. DDB conceived of and coordinated the study, and drafted background and discussion sections. All authors read and approved the final manuscript.

## Authors' information

SCN, SKN and EM were undergraduate students at the time this study was carried out. SCN and SKN have continued to medical school, while EM is now a graduate student in the Dept. of Bioengineering at the University of Utah. JDB is Dean of Undergraduate Education. DDB is Professor of Physiology and Biophysics.
